# Dehydration Time Effect on Tooth Color Measurement: An *In Vitro* Study

**DOI:** 10.1055/s-0041-1741377

**Published:** 2022-03-13

**Authors:** Yasmine T. Ahmed, Fahad A. Almutairi, Shaima A. Alomran, Nourah M. Alkhayatt, Shahad A. Alsulaiman, Salma Y. Alohali, Albatool A. Alhamdi

**Affiliations:** 1 Department of Restorative Dentistry, College of Dentistry, Riyadh Elm University, Riyadh, Kingdom of Saudi Arabia; 2 Private Practice, Riyadh, Kingdom of Saudi Arabia; 3 Department of Dentistry, College of Dentistry, King Saud University, Riyadh, Kingdom of Saudi Arabia

**Keywords:** tooth color, dehydration time, shade selection, color measurement

## Abstract

**Objectives** Esthetics have become a primary influencing factor for patient satisfaction, thus dental restorations shade selection is critical, as it should closely resemble a healthy tooth. During various dental procedures, teeth are subjected to dehydration. The commonly used shade guides are being replaced with electronic color measurement devices for more precise measurements. The aim of this study was to evaluate the effect of dehydration time on tooth color measurement using a spectrophotometer.

**Materials and Methods** Study sample is represented by 20 extracted caries-free maxillary central incisors, which were obtained from several private clinics in Riyadh, Saudi Arabia, and soaked in normal saline for 24 hours. The VITA Easyshade Advance 4.0 spectrophotometer was used to measure the color at different areas of the tooth (cervical, middle, and incisal thirds) at three time intervals (baseline, after 1 hour of dehydration, and after 2 hours of dehydration).

**Statistical Analysis** All color coordinates across the different areas of each tooth at the incisal, middle, and cervical thirds descriptive statistics of mean (standard deviation) values were calculated and were compared at the different time intervals at baseline, after 1 hour of dehydration, and after 2 hours of dehydration. Finally, the color change value ∆E was calculated using the formula ∆E* _ab_ = √ (L _2_ ∗ − L _1_ ∗) ^2^ + (a _2_ ∗ − a _1_ ∗) ^2^ + (b _2_ ∗ − b _1_ ∗) ^2^ .

**Results** The color difference ∆E showed statistically significant changes at different time intervals: at baseline, after 1 hour of dehydration, and after 2 hours of dehydration ( *p* < 0.001). Hue had statistically significant changes between 1 hour and 2 hours of dehydration ( *p* = 0.002). Chroma value also showed statistically significant changes ( *p* < 0.001) in all time intervals.

**Conclusion** By using VITA Easyshade Advance 4.0 spectrophotometer, it was indicated that the tooth color measurements were significantly affected by dehydration time, and tooth shade appeared lighter due to changes in the refractive indices as air replaces the interprism spaces within the enamel. Tooth color measurements for shade selection should be taken as soon as possible to limit dehydration effect and ensure a more accurate shade selection for an enhanced esthetic result.

## Introduction

Recently, dental demands shifted from functional dental treatments to more esthetic, especially with more awareness and care toward individual oral health. 1 Social media has also proven to affect the perception and demand of dental esthetics within the population. 2 3 4 Dental treatments including orthodontic treatment, prosthetics, and esthetic restorations are all of high importance. Dental restorations, in particular, are more technically sensitive especially in anterior teeth, due to the large variety of factors such as teeth color, shape, and position which are affected by patients' preferences and sociodemographic; gender, age, education level, and previous dental treatments affect patient satisfaction. 5 Many studies showed that ∼80% of patients were dissatisfied with their restoration due to the color as they compared it with adjacent teeth. 6 Human teeth show unique opalescence, translucency, and fluorescence, which should be restored by esthetic restorative materials. 7 Therefore, dental restorations should mimic the color and optical properties of healthy teeth, especially in the anterior area. 8

Accurate measurement of tooth color is essential for a successful aesthetic result. Dentists must have adequate training in selecting tooth color and are aware of the scientific aspects as well as the factors influencing tooth color and shade taking. Color vision is a result of a complex process of stimulation, sensation, and perception. Through optical phenomena, light is absorbed, reflected, and transmitted by the tooth surface. 9 Color perception is influenced by three variables: the light source, the object, and the observer's eyes and brain. 10 Object color is the result of a perception of light reflected or scattered from its surface. Accordingly, the tooth color is a result of the perception of light scattered within the tubules of dentin and hydroxyapatite crystals of the enamel. 11 Factors affecting the observer include eye fatigue, aging, emotional and physiological issues, and light conditions of the surrounding environment will affect visual shade selection. 12

A variety of tools are used to determine color, including shade guides, spectrophotometers, colorimeters, spectroradiometers, and digital image analysis. 13 A shade guide is a set of colored tabs which are used as a standard resembling teeth structures. 14 It is a quick, easy, and cost-effective way to choose shades, but it is considered subjective and inconsistent. Several factors can influence visual shade selection, such as the lighting, color acuity, and eye fatigue. 15 These undesirable conditions can be overcome by using sophisticated instruments such as spectrophotometers and colorimeters.

A spectrophotometer measures and records the amount and spectral composition of light reflected from the tooth. 16 17 It is the most accurate and flexible instrument used for color matching. 18 Data are quantified and easily collected, but it is mostly used in research due to its complexity and high costs. 19

Dehydration of the teeth can increase enamel opacity, making them seem whiter. 10 20 Interprism spaces become filled with air instead of water, so light cannot scatter between crystals. 21 Loss of translucency on dehydration results in more reflection, which masks the underlying color of dentine, which makes it appear lighter. 22 23 Most dental procedures cause some dehydration of teeth. 20 It is recommended to record color before any restorative procedure. 24 Mismatches between restorations and natural teeth may result in remakes and increased expense.

There is limited quantitative evidence in the current literature about the influence of tooth dehydration on color measurements and shade selection mismatch. The primary aim of this study was to assess the effect of dryness and dehydration on tooth color using a spectrophotometer.

## Materials and Methods

The present study was conducted at the Riyadh Elm University (REU) from June 13, 2021, to August 16, 2021. The ethical approval was obtained from the Institutional Review Board of REU with approval number SRP/2021/457/475. Twenty freshly extracted maxillary central incisors were collected from several private clinics in Riyadh, Saudi Arabia, and immediately soaked in normal saline for 24 hours. The selection criteria for the teeth were the normal anatomical shape of the tooth and absence of visible defects, restorations, stains, excessive abrasion, and caries on the labial surfaces. They were extracted due to periodontal issues.

### The Spectrophotometric Analysis

The color measurements were performed using the VITA Easyshade Advance 4.0 spectrophotometer. The experiment was conducted in a dark room to minimize changes in light conditions because any change can affect measurements due to the translucent nature of tooth substance. 25 The spectrophotometer was calibrated and used according to the manufacturer's instructions before each set of measurements and was used with the “Tooth Areas” setting to measure color at the cervical, middle, and incisal areas. An infection control shield was used to prevent any contamination and damage of the optical fibers. Spectrophotometer measurements were performed for all teeth by a single operator, placing the probe perpendicular to the tooth surface, pressing the measurement button, and holding the probe tip against the tooth until two quick “beeps” can be heard to indicate completion of the measurement. A total of three measurements were performed, baseline spectrophotometric measurements were obtained (cervical, middle, and incisal), after that, the teeth were dried at room temperature for 1 hour then 2 hours, respectively. Further spectrophotometric measurements were performed (cervical, middle, and incisal). After all the teeth are dehydrated naturally with air dry. The color data from Easyshade recoded by using the International Commission on Illumination (CIE) L*a*b* coordinates, Chroma (c), hue (h). The color difference (∆E) were calculated between two different dehydration times by using: ∆E* _ab_ = √ (L _2_ ∗ − L _1_ ∗) ^2^ + (a _2_ ∗ − a _1_ ∗) ^2^ + (b _2_ ∗ − b _1_ ∗) ^2^ . The CIE laboratory identifies light wavelengths as numbers represented in three coordinates (L = lightness, a = green and red, and b = blue and yellow). The L coordinate represents the lightness and darkness of the specimen, the greater the L*, the lighter the specimen. The a coordinate measures the chroma within the red and green axis. A positive a value corresponds to the amount of redness, whereas a negative a value relates to the amount of greenness. And the b* coordinate measures of the chroma along the yellow and blue axis, a positive b value relates to more yellowness, and a negative b value relates to more blueish the color. 26

### Statistical Analysis

Descriptive statistics of mean (standard deviation) values were calculated for all the color coordinates at a different time interval and compared across cervical, middle, and incisal thirds of the teeth. A color change value ∆E was obtained ∆E* _ab_ = √ (L _2_ ∗ − L _1_ ∗) ^2^ + (a _2_ ∗ − a _1_ ∗) ^2^ + (b _2_ ∗ − b _1_ ∗) ^2^ . Similarly, hue and chroma values were compared across different areas. All the data collected on color measurements were entered into the statistical package for social sciences (IBM-SPSS version 25, Armonk, New York, United States), and analysis was performed. A *p* -value of less than <0.05 was considered significant for all the statistical tests. The null hypothesis of this study is that there is no difference between tooth shade before and after dehydration.

## Results

**Fig. 1:**
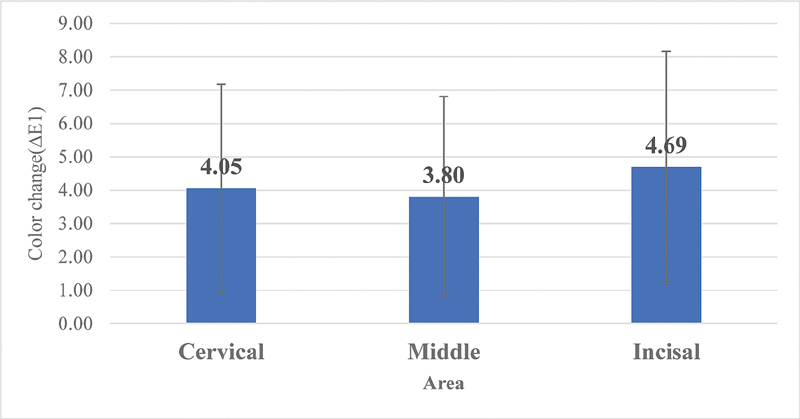
Color change from baseline to after 1 hour in different areas ( *F* = 0.408, *p* = 0.667).

**Fig. 2:**
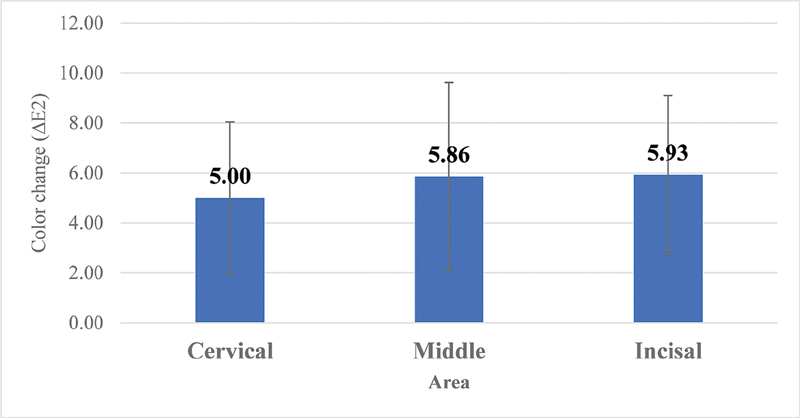
Color change from baseline to after 2 hours in different areas ( *F* = 0.483, *p* = 0.619).

**Fig. 3:**
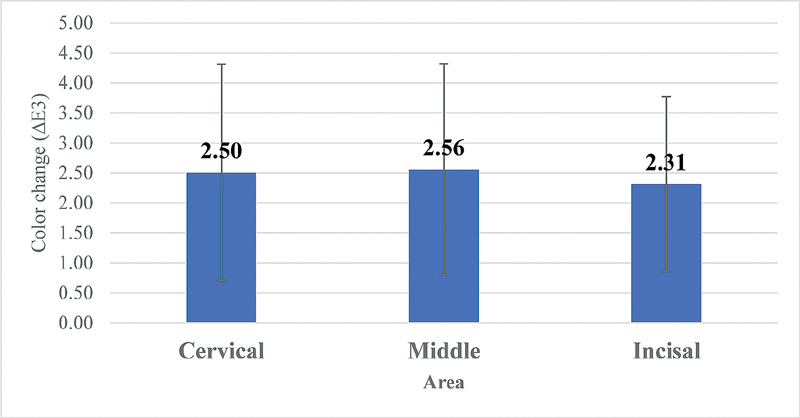
Color change from 1 hour to 2 hour in different areas ( *F* = 0.119, *p* = 0.888).

The analysis of variance (ANOVA) indicated that there were no statistically significant changes over time from the baseline, after 1 hour, and 2 hours of dehydration between different areas of the tooth surface: cervical, middle, and incisal, respectively, for color coordinates: L, C, h, a, b as shown in Table 1 . From baseline to 1 hour of dehydration, there were no significant changes ( *p* = 0.667) ( Fig. 1 ). When comparing baseline to 2 hours of dehydration, there were no significant color changes ( *p* = 0.619) ( Fig. 2 ), and finally, when comparing 1 hour of dehydration to 2 hours still no changes were found ( *p* = 0.888) ( Fig. 3 ). However, there were statistically significant changes in the color difference ∆E with different time intervals: between baseline, after 1 hour, and 2 hours of dehydration.

**Table 1:** Descriptive statistics for color coordinates: L, C, h, a, b between different areas of the tooth surface: cervical, middle, and incisal, at different time intervals

The color coordinates at different time intervals							
Time	Color coordinates	Cervical		Middle		Incisal	
		Mean	SD	Mean	SD	Mean	SD
Baseline	L0	79.15	6.24	81.27	5.71	81.09	4.60
	C0	19.21	5.54	17.65	6.37	17.28	7.17
	h0	94.02	5.45	96.48	5.47	93.85	13.88
	a0	−1.26	1.30	−1.30	1.34	−1.45	1.60
	b0	19.21	5.52	17.59	6.38	17.26	7.15
1 h	L1	81.61	6.10	84.27	5.11	85.10	4.76
	C1	20.91	5.19	19.14	6.20	18.53	7.41
	h1	92.27	4.54	95.21	5.46	95.37	7.43
	a1	−0.72	1.20	−1.02	1.21	−0.98	1.41
	b1	21.31	5.80	19.12	6.21	18.52	7.41
2 h	L2	82.41	6.79	86.05	5.69	85.80	4.19
	C2	21.78	5.54	20.13	6.33	19.68	7.57
	h2	91.08	4.78	94.16	4.92	93.76	6.13
	a2	−0.43	1.41	−0.70	1.26	−0.77	1.42
	b2	21.55	5.51	20.06	6.29	19.63	7.55

Abbreviations: a, chromaticity coordinate for red–green; b, chromaticity coordinate for yellow–blue; C, chroma value; h, hue value; L, lightness value; SD, standard deviation.

The ANOVA test indicated that there were statistically significant changes in the ∆E ( *p* < 0.05) shown in Table 2 , as follows:

**Table 2:** Color change value at different time intervals

Paired differences in color change values (∆E)							
Color change	Mean	SD	Standard error mean	95% confidence interval of the difference		Time	*p* -Value
				Lower	Upper		
∆E1 − ∆E2	−1.42	2.23	0.29	−1.99	−0.84	−4.912	<0.001
∆E1 − ∆E3	1.72	3.12	0.40	0.92	2.53	4.279	<0.001
∆E2 − ∆E3	3.14	2.90	0.37	2.39	3.89	8.371	<0.001

Abbreviations: ∆E1, color difference at baseline; ∆E2, color difference after dehydration for 1 hour; ∆E3, color difference after dehydration for 2 hours; SD, standard deviation.

1. From baseline to 1 hour of dehydration: ∆E1 − ∆E2 ( *p* < 0.001)
2. From baseline to 2 hours of dehydration: ∆E1 − ∆E3 ( *p* < 0.001)
3. Between 1 hour and 2 hours of dehydration: ∆E1 − ∆E3 ( *p* < 0.001)

- ∆E1: at baseline
- ∆E2: after dehydration for 1 hour
- ∆E3: after dehydration for 2 hours.

In addition, results showed statistically significant changes in hue only between 1 hour and 2 hours of dehydration ( *p* = 0.002) in Table 3 . Chroma value also showed statistically significant changes over time of dehydration from the baseline, after 1 hour, and 2 hours, respectively ( *p* < 0.001) in all time intervals as shown in Table 4 .

**Table 3:** Paired differences in hue values at different time intervals

Paired differences in hue values at different time intervals							
Hue	Mean	SD	Standard error mean	95% confidence interval of the difference		*t* -Value	*p* -Value
				Lower	Upper		
Between baseline and 1 h	0.50	7.82	1.01	−1.52	2.52	0.493	0.624
Between baseline and 2 h	1.78	7.95	1.03	−0.27	3.84	1.737	0.088
Between 1 h and 2 h	1.29	3.05	0.39	0.50	2.07	3.259	0.002

Abbreviation: SD, standard deviation.

**Table 4:** Paired differences in chroma values at different time intervals

Paired differences in hue values at different time intervals								
Chroma value	Mean	SD	Standard error mean	95% confidence interval of the difference		*t* -Value	df	*p* -Value
				Lower	Upper			
Between baseline and 1 h	−1.49	1.50	0.19	−1.87	−1.10	−7.671	59	<0.001
Between baseline and 2 h	−2.49	2.12	0.27	−3.03	−1.94	−9.106	59	<0.001
Between 1 h and 2 h	−1.00	1.36	0.18	−1.35	−0.65	−5.685	59	<0.001

Abbreviations: df, degree of freedom; SD, standard deviation.

## Discussion

In this study, Vita Easyshade Advance 4.0 spectrophotometer was used to record tooth color changes in response to different dehydration time due to the limited number of studies that have investigated the correlation between dehydration and tooth shade. Electronic shade selection methods such as spectrophotometric shade analysis were found more accurate, reliable, and reproducible compared with the conventional method of human visual shade assessment. 27 28 29 The CIELAB system was selected to measure color variations due to its ability in recording minor color variations. 30 The spectrophotometer and the CIE color system provided precise color difference evaluations that surpassed the visual evaluations limitations. 31 Differences of CIE L*a*b* values between (cervical, middle, and incisal) regions were clinically and statistically not significant in contrast to previous researches in which there was an overall gradation in color from the cervical region which is most opaque to the incisal region becoming more translucent 32 as shown in Table 1 . This could be a result of the underlying absorption pattern of the tooth as dentin and not only the enamel. 33

To determine the change in tooth color due to dehydration, tooth shade was measured at the baseline, after 1 hour, and 2 hours. The results indicated a significant change in tooth color after 1 hour and 2 hours due to dehydration as shown in Table 2 . At the different time intervals, the authors note that ∆E value increases gradually, as it can be seen from baseline to 1 hour (∆E1 − ∆E2 = − 1.42), when compared with the mean from baseline to 2 hours (∆E1 − ∆E3 = 1.72) and from 1 hour to 2 hours (∆E2 − ∆E3 = 3.14). This interpretation validates the results of Burki et al, in which there were statistically significant changes due to dehydration after 10 and 30 minutes. 24 After the statistical analysis, it was indicated that there is a significant rejection of the null hypothesis which stated there is no change in the tooth color associated with dehydration; however, the lightness (L) which represents the amount of light reflected by an object, in this case the tooth surface, compared with a pure white diffuser (an object that only reflects light rays) and a black absorber (an object that only absorbs light rays), 34 showed no statistically significant changes, even though it is known that dehydration leads to increase in the opacity of the tooth making it lighter. 10 20 As mentioned earlier, spectrophotometers reproduce accurate measurements compared with visual assessment of tooth color, but it can give incorrect results if the investigator is not trained with the instrument resulting in repositioning errors, as this could be the case in this study. To limit the minor repositioning error during the teeth assessment, it is important to orient the spectrophotometer probe in the same position each time. A prior study noted the importance of using custom-made positioning jigs for each subject to prevent the arbitrary placement of the probe. 24 Hue is the quality that differentiates one color from another. 35 The change in hue in this study was significant; we found a significant change between 1 hour and 2 hours of dehydration where *p* = 0.002 as shown in Table 3 . The significance is possible because of a change in the reflectance spectrum of the dehydrated enamel. 36 Chroma is a major determinant of color, defined as the saturation of a specific color. 37 It changed significantly with dehydration as shown in Table 4 . This significance is mainly due to the change of the refractive index within both surfaces leading to more light scatter, thus appearing lighter. 22

The spectrophotometer (Vita Easyshade Advance 4.0) is considered an extremely reliable method to have accurate shade measurements and that is the reason we have used it in this study. 38 However, according to Judeh and Al-Wahadni, further device improvements and software upgrades would help dentists to select better shades. 39

Limitations of the study include long time intervals between readings to measure the dehydration effect. Additionally, a small sample size was used in the study, which may have significant difference on the outcome when applied to a larger sample. Furthermore, the experiment conducted on the sample was performed under ideal conditions, which are not present in an actual clinical setting. Finally, the teeth used were not specific as they were randomly collected from private clinics, so selected teeth had great diversity of age, gender, and ethnicity; hence, tooth shades collected results were affected. Additionally, the accuracy of the VITA Easyshade Advance 4.0 device might have some minor defects that occur in electronic devices.

We recommend in future studies to decrease dehydration time intervals, since teeth in the operative setting are subjected to continuous dehydration and rehydration effects. 36 For future *in vitro* studies increasing the sample could be beneficial to validate the results. Sample selection could be from a specific age and ethnicity group with similar initial tooth shade to make the study more precise and reveal a clearer picture regarding the different dehydration patterns. 22 Additional recommendations include clinical application of the study with the use of a rubber dam to evaluate the dehydration effect on teeth in clinical setting.

## Conclusion

Dehydration time dramatically affects tooth color measurements, and the spectrophotometer Vita Easyshade Advance 4.0 proved to be reliable in detecting tooth color changes at different durations of dehydration. Postdehydration teeth appear brighter due to the change in the refractive index caused by air replacing the interprism spaces within the enamel; therefore, color measurements for shade selection should be taken as soon as possible to ensure accuracy of shade selection and to provide a successful aesthetic result. Dentists must have proper training in tooth color selection and be aware of the scientific components as well as the factors that influence tooth color and shade taking. Incompatibility between restorations and natural teeth will result in an increased chance of remakes and higher expenses, as patients will not be satisfied with the results.
